# Acoustofluidic scanning fluorescence nanoscopy with a large field of view

**DOI:** 10.1038/s41378-024-00683-8

**Published:** 2024-05-10

**Authors:** Geonsoo Jin, Neil Upreti, Joseph Rich, Jianping Xia, Chenglong Zhao, Tony Jun Huang

**Affiliations:** 1 Thomas Lord Department of Mechanical Engineering and Materials Science, Duke University, Durham, NC 27708 USA; 2 Department of Biomedical Engineering, Duke University, Durham, NC 27708 USA; 3 The MITRE Corporation, McLean, VA 22102 USA

**Keywords:** Micro-optics, Nanophotonics and plasmonics

## Abstract

Large-field nanoscale fluorescence imaging is invaluable for many applications, such as imaging subcellular structures, visualizing protein interactions, and high-resolution tissue imaging. Unfortunately, conventional fluorescence microscopy requires a trade-off between resolution and field of view due to the nature of the optics used to form the image. To overcome this barrier, we developed an acoustofluidic scanning fluorescence nanoscope that simultaneously achieves superior resolution, a large field of view, and strong fluorescent signals. The acoustofluidic scanning fluorescence nanoscope utilizes the superresolution capabilities of microspheres that are controlled by a programmable acoustofluidic device for rapid fluorescence enhancement and imaging. The acoustofluidic scanning fluorescence nanoscope resolves structures that cannot be resolved with conventional fluorescence microscopes with the same objective lens and enhances the fluorescent signal by a factor of ~5 without altering the field of view of the image. The improved resolution realized with enhanced fluorescent signals and the large field of view achieved *via* acoustofluidic scanning fluorescence nanoscopy provides a powerful tool for versatile nanoscale fluorescence imaging for researchers in the fields of medicine, biology, biophysics, and biomedical engineering.

## Introduction

Fluorescence microscopy has become an indispensable technique in the fields of biology and medicine^1^ with applications ranging from microscale imaging of live cells to nanoscale imaging of DNA sequencing protocols^2,3^. However, due to the structures of the objective lens used in conventional fluorescence microscopy, a trade-off is required between the resolution and field of view. A higher-resolution image from a conventional fluorescence microscope can be achieved by using an objective lens with higher magnification (typically also with a higher numerical aperture), but it is typically at the cost of a reduced field of view. One effective approach for increasing the resolution while maintaining a large field of view involves the use of scanning dielectric microspheres^4–13^. When the dielectric microsphere has a refractive index higher than that of the outer medium, the propagated light is focused from the inside of the microsphere, and a highly localized electromagnetic beam is generated near its surface, which is known as a photonic nanojet, and it provides superresolution imaging below the diffraction limit.

Photonic nanojets have been utilized to enhance the resolution of both white light^14–25^ and fluorescence microscopic imaging^26–28^. For example, an optical fiber probe was combined with a microsphere for manipulation and detection of individual sub-100 nm fluorescent nanoparticles^26^. Moreover, a 20 nm fluorescent nanoparticle was also detected with a microsphere array in a microfluidic manner^27^. A semi-opened microwell on a microsphere also captured target samples and amplified the fluorescence signal via the photonic nanojet effect^28^. However, these studies had limited detection areas due to static microsphere imaging or fixed microsphere conditions. By incorporating a dynamic scanning element, such as an AFM cantilever^29,30^, mechanical stage movements^31,32^, optical tweezer methods^33^, or acoustofluidics^34,35^, both high resolution and a large field of view can be achieved. Among these methods, acoustofluidic manipulation is advantageous because it has a programable process, vast particle size manipulation range, and contactless manipulation nature^36–53^. Recently, we demonstrated that acoustically driven microspheres acted as scanning superlenses to rapidly and simultaneously achieve a large field of view and high resolution in a white-light microscope^34,35^. However, application to fluorescence microscopy has yet to be explored.

In this article, we introduce an enhanced acoustofluidic scanning nanoscope for fluorescence imaging and amplification, which is supported by quantitative analyses. By utilizing visibility metrics and resolution benchmarks, we demonstrated that under identical imaging conditions, an acoustofluidic fluorescence scanning nanoscope resolved structures that remained indistinct when imaged with a conventional fluorescence microscope utilizing the same objective lens. This method enhanced the fluorescent signal by a factor of ~5 and maintained the field of view, offering a quantifiable improvement in imaging resolution.

## Results and discussion

### Configuration of the acoustofluidic scanning fluorescence nanoscope

**Fig. 1:**
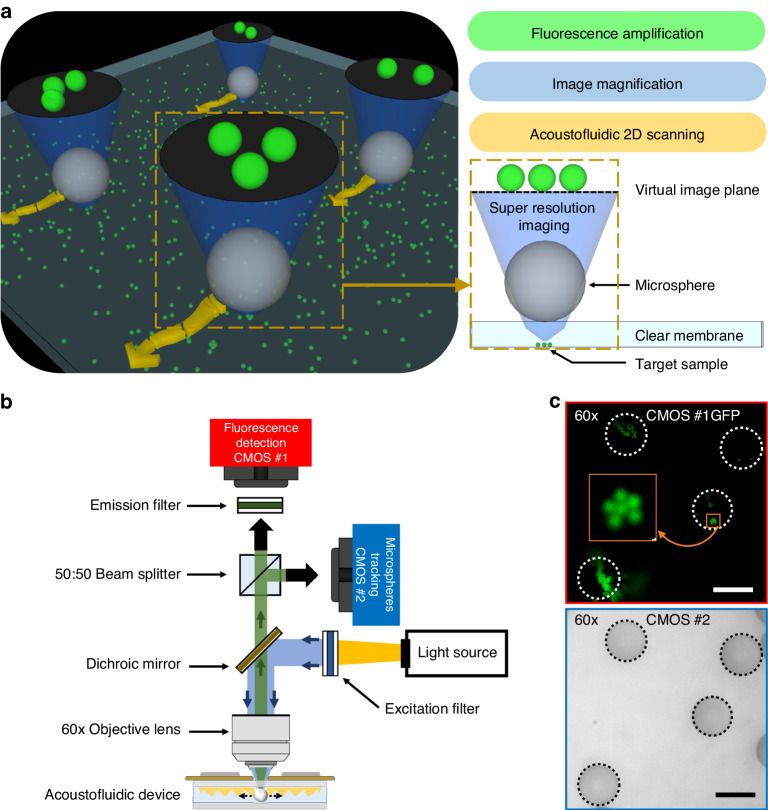
Mechanism of the acoustofluidic scanning fluorescence nanoscope. **a** 3D schematic of the system. A hard PDMS membrane on the target sample achieved the desired focal distance and provided high-resolution images, as shown by the yellow box in the 2D schematic on the right. **b** Schematic of the optical setup. A 50:50 beam splitter delivered images into two cameras for both fluorescence detection (camera #1, red box) and microsphere tracking (camera #2, blue box). **c** Enhanced fluorescent amplification of 500 nm fluorescent nanoparticle images (Camera #1, red box) through microspheres and microsphere particle tracking (Camera #2, blue box). Camera #2 was focused on the center of the microspheres, as shown in the blue box. Only camera #1 was connected to an emission filter. Scale bars are 20 µm

Figure 1a shows a 3D schematic of the acoustofluidic scanning fluorescence nanoscope. Superresolution imaging was achieved when a microsphere was placed on the target sample, as shown in the yellow dotted box in Fig. 1a. The sample consisted of fluorescent nanoparticles that were drop-cast on a cover glass. The fluorescent particles were then covered by a thin layer of PDMS film to lock their positions on the cover glass and avoid drifting during the imaging process. A large field-of-view image was achieved by stitching the superresolution images from the scanning microspheres. Scanning of the microspheres was achieved by activating a propagating acoustic wave following the same method we used previously^34,35^ or by counterpropagating the acoustic waves that are demonstrated in this work. The advantage of using counterpropagating acoustic waves is that we can easily control the direction of the scan, which cannot be achieved with a propagating acoustic wave.

Figure 1b shows the optical configuration of the system. The specifications of the required components can be found in the experimental section. A white light source combined with a blue bandpass filter was used to illuminate the sample, and the light was passed through a dichroic mirror and focused through a ×60 objective lens. The green fluorescent light from the nanoparticles was collected with a CMOS camera (shown as a red camera #1 in Fig. 1b) through the same objective lens combined with a green bandpass filter (denoted as the emission filter in Fig. 1b). A 50:50 beam splitter was placed in the light path to add a second camera (shown as a blue camera #2 in Fig. 1b) to the system without the emission filter to track the position of each microsphere. The positions of the two cameras (#1 and #2) were adjusted so that both the sample and the microsphere were imaged simultaneously with camera #1 and camera #2, respectively. The red box in Fig. 1c shows an image of 500 nm fluorescent nanoparticles on camera #1 that were imaged with four microspheres. The four white dashed circles indicate the boundaries of the four microspheres. Note that the four microspheres are invisible in the fluorescence image on camera #1. In contrast, they were clearly imaged on camera #2, as shown in the blue box in Fig. 1c. The images of the microspheres on camera #2 are critically important for determining the exact position of the fluorescence image from each microsphere so that they can be stitched correctly to form the final large field-of-view image. The position of each microsphere was obtained from the image on camera #2 by using a circle-finding algorithm in the imaging process. This position information from camera #2 was assigned to the fluorescence image on camera #1. As a result, this dual-camera configuration allowed us to construct a high-resolution fluorescence image with a large field of view by precisely stitching the fluorescence images from each microsphere.

### Improved resolution and enhanced fluorescent signals with microspheres

**Fig. 2:**
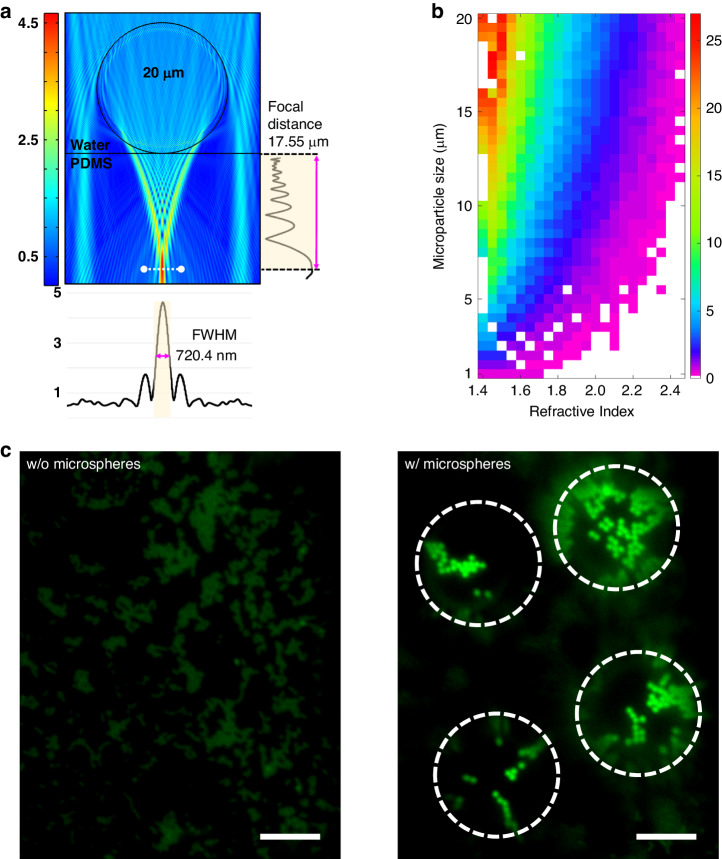
Simulation and experimental results for the photonic nanojets. **a** Finite element method (FEM) simulation results for a 20 µm polystyrene microsphere (*n* = 1.58) in water (*n* = 1.33) with a hard PDMS (*n* = 1.41) film on the bottom as the outer medium. The vertical graph shows the focal distance from the bottom of the microsphere to the PDMS membrane. The horizontal graph represents the focused photonic nanojets and their full-width half-maxima. **b** FEM simulation of the focal distance map as a function of the microsphere diameter and refractive index. The red color (25 µm focal distance) to purple color (3 µm focal distance) show each focal distance depending on the size of the microsphere and the refractive index. **c** Microscopy images showing the microsphere magnification capability. The left panel shows an image captured without microspheres. The right panel shows microsphere magnification at the same regions of interest. The target sample comprised 500 nm green fluorescent nanoparticles, and the thickness of the hard PDMS membrane was 7 µm. The scale bar is 10 µm

Figure 2a shows the simulated electric field distribution of a 20 µm polystyrene microsphere (refractive index *n* = 1.58) determined *via* finite element methods. The microsphere sits on a hard PDMS (*n* = 1.41) with a surrounding medium of water (*n* = 1.33), as in the experiment. Light with a wavelength of 488 nm was used to excite the green fluorescence. The simulation confirmed that the light was well focused by the microsphere to a spot with a full width at half maximum (FWHM) of 720 nm and a distance of 17 µm away from its surface (defined as its focal length), as shown on the vertical graph in Fig. 2a. The focal length can be changed by choosing microspheres with different sizes or refractive indices. Figure 2b shows the color map of the focal length as a function of the microsphere diameter and refractive index, which can be used as a guide to select the right microspheres for the desired focal length. Changing the focal length of a microsphere changes the position of the virtual image from the microsphere, which must be compensated by adjusting the position of the objective lens for clear imaging. Supplementary Fig. S1 contains the simulation results.

Figure 2c shows experimental fluorescence images of the same sample without and with a microsphere. The sample consisted of aggregated fluorescent nanoparticles (500 nm in diameter) that were sandwiched between a 7 µm-thick PDMS film and a glass substrate (see the “Experimental section”). The two images shown in Fig. 2c were taken in the same region of interest with the same settings, such as the light intensity and camera exposure time. The presence of the microspheres provided increased resolution of the system and enhanced fluorescence signals.

**Fig. 3:**
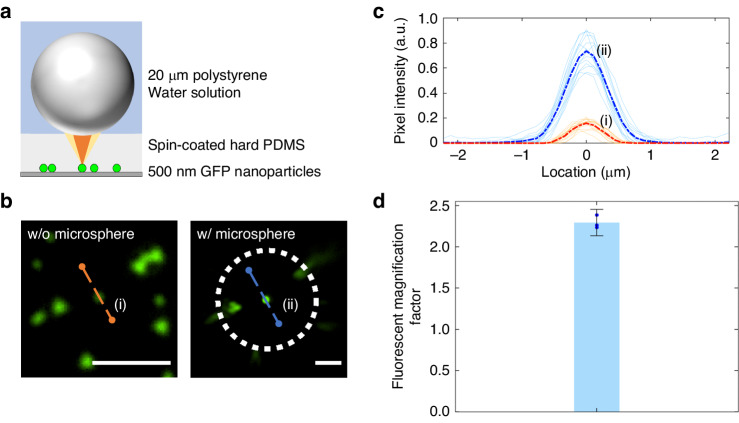
Fluorescence signal amplification with microspheres. **a** Schematic of fluorescence amplification with microsphere imaging. Green fluorescent nanoparticles (500 nm) were spread on a glass substrate. Then, a hard PDMS membrane was applied by a spin-coating process. Polystyrene microspheres (20 µm) and a deionized water solution were applied to the target sample. **b** Microscopy images of microsphere magnification and fluorescence amplification in a 7 µm thick layer of hard PDMS. Since the microsphere magnified the image, it is larger than that seen without microspheres, and the different magnifications of each row of images were indicated by altering the lengths of the scale bars. Scale bar: 5 µm. **c** Fluorescence amplification profile without and with microspheres. The blue and red profiles show the average pixel profile intensities of 15 samples without microspheres (i) and with microspheres (ii), as shown in Fig. 3b. **d** Bar graph displaying the average fluorescence magnification factor with a microsphere. The error bars represent ±2 STDEV. The data processing procedure is explained in Fig. S6

The fluorescence amplification results are illustrated in Fig. 3. Figure 3b shows images of fluorescent nanoparticles obtained with and without a microsphere. The presence of a microsphere on top of the sample clearly enhanced the fluorescence signal. Figure 3c shows the fluorescence profiles of 15 samples with (light blue lines) and without microspheres (light red lines). The blue dashed line and the red dashed line show the average intensities of the 15 samples. The fluorescence enhancement factor, which is defined as the ratio of the average intensity of the fluorescence with a microsphere to that without a microsphere, was ~5 (Fig. 3c). Finally, Fig. 3d shows a quantifiable increase in magnification observed when using a microsphere. Although there were slight variations for the different magnifications, the increases were consistently >2×. More detailed analyses of the increased magnification and images of the results can be seen in Fig. S6.

### Bidirectional acoustofluidic scanning of microspheres

**Fig. 4:**
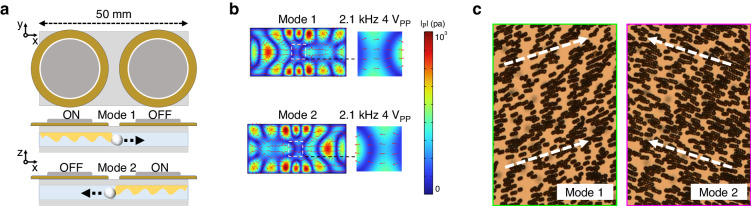
Acoustofluidic scanning device for bidirectional microsphere manipulation. **a** Schematic of the bidirectional acoustofluidic manipulation device, which incorporated two circular piezoelectric transducers bonded onto a cover glass with a thickness of 150 µm. The space between the two transducers was 6 mm. Mode 1 operates by pushing microspheres to the right; in contrast, mode 2 pushes them to the left. **b** Simulation results of the bidirectional acoustofluidic device operating in modes 1 and 2. The working frequency was 2.1 kHz, and the amplitude was 4 V_PP_. The bidirectional acoustic streams observed with the two modes are shown in the green and pink boxes in the working area. **c** Stacked microscopy images from the space between two transducers showing that the 20 µm microspheres were manipulated bidirectionally by acoustic streaming in each operation mode

To perform efficient 2D scanning over a large field of view, we designed and fabricated a bidirectional acoustofluidic scanning device. As shown in Fig. 4a, two circular piezoelectric transducers were bonded onto a cover glass with a thickness of 150 µm. A distance of 6 mm was established between the two transducers, meaning that the detection area size was sufficient for microscopic imaging even under the largest field of view with a 4x objective lens, which is typically 4 mm in diameter. This bidirectional acoustofluidic scanning design operates in two modes programmed in a MATLAB interface that allowed us to control the direction of acoustic wave propagation and the scan direction of the microspheres separately.

Figure 4b shows the simulated acoustic energy distribution obtained with modes 1 and 2. The simulation assumed that an electric wave of 2.1 kHz with a peak-to-peak voltage of 4*V*_PP_ was applied to the transducer. The insets show the acoustic energy distributions of the two piezoelectric transducers, with the white arrows showing the directions of the acoustic energy flow. Notably, the acoustic pressure amplitude across the chip changed significantly within a region spanning just 50 mm, which was much smaller than the wavelength of 714 mm. This pronounced local variation was attributed to interactions among the chip structural elements. Specifically, this arose from the displacement distribution, which was influenced by the free boundary condition of the cover glass and coupling between the transducer and the cover glass. This condition introduced constraints that altered the wave patterns, leading to an unexpected intensity profile within a relatively short scale. These interactions led to a displacement distribution that differed from conventional expectations based on wavelength alone. An in-depth explanation of the underlying physics can be found in Supplementary Fig. S5, which provides a graphical representation and a comprehensive analysis of the displacement distribution and its consequences on the acoustic pressure amplitude within the chip. Figure 4c shows stacked experimental images of microsphere movement with mode 1 and mode 2. The microspheres were scanned from left to right and from right to left in mode 1 and mode 2, respectively (see Supplementary Video SV1). Microparticle floating was observed at a *V*_PP_ > 4, as shown in Supplementary Fig. S2, so 5*V*_PP_ was applied with a frequency of 2.1 kHz and a 0.2 s interval burst during the procedure. Compared to the acoustic scan obtained with one transducer in our previous work^34,35^, this bidirectional scanning design provided more degrees of freedom to scan the microspheres and achieve high-resolution, large-field-of-view images.

### Image distortion correction and large-field-of-view imaging

**Fig. 5:**
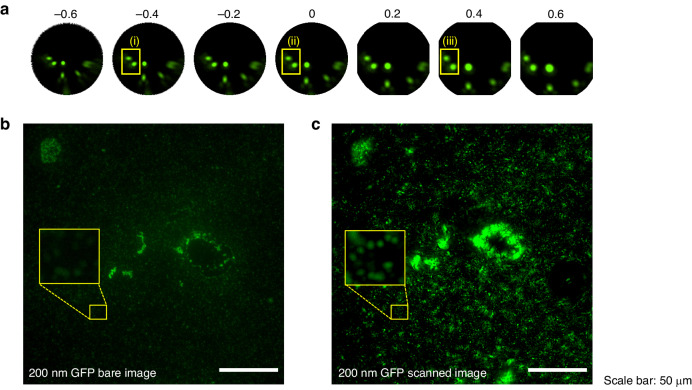
Image restoration of lens distortion and large field-of-view imaging with the acoustofluidic scanning fluorescence nanoscope. **a** Image distortion restorations with different parameters. A parameter of 0 indicates the original image. A negative parameter resulted in the correction of barrel distortion, and a positive parameter resulted in the correction of a pincushion distortion. A barrel-type distortion was observed in the small yellow box in the original image (ii). The −0.4 adjusted image (i) displays distorted nanoparticles, and the 0.4 adjusted image (iii) shows round nanoparticles due to the distortion correction. **b** A bare image of 200 nm nanoparticles deposited onto the sample surface. **c** An acoustofluidic scanned image of the same sample. A total of 141 scanned images were processed to create the final scanned image. The yellow boxes indicate the same region of interest in the sample. The scale bar is 50 µm

Recently, various techniques for restoring distorted images have been introduced^54–57^. In this article, the off-axis fluorescence image from a microsphere showed large image aberrations, as manifested in the image (the comet-like tails in the images located at the edge of the microsphere) shown in Fig. 3b. Since each of the distorted images came from a single nanoparticle, the distortion was corrected with a MATLAB algorithm that allowed us to adjust different lens distortions by changing the value of an input parameter. Figure 5a shows the effects of different parameters on image correction. The original image (ii) was corrected by assigning a positive value of 0.4, as shown in the image (iii) in Fig. 5a. In contrast, a negative value of -0.4 deteriorated the image. To verify this image correction process, a more identifiable sample (a line grating) was imaged with a microsphere, and the image of the grating was recovered by using this image correction process, as shown in Supplementary Fig. S3.

After confirming the image correction process, a large field-of-view fluorescence image was obtained by merging the images from the scanning microspheres and applying the image-correction algorithm. A Python image processing tool was used to merge the images. The location data of each image were first generated by camera #2 and then applied to the image data in camera #1. Each microsphere image was then cropped and pasted to create the final scanned image. This procedure was executed recursively until every region was covered.

Figure 5b shows an image of 200 nm green fluorescent nanoparticles obtained directly from a microscope without the use of microspheres. Figure 5c shows the same image obtained with scanning microspheres, which contains a nanoparticle sample with stronger fluorescence intensity. The yellow boxes in Fig. 5b, c indicate a much higher intensity in the same region of interest in the scanned image. To obtain a fully scanned image (200 × 200 µm field of view), we acquired and processed 141 microsphere images within 5 min (1 min of image acquisition, 4 min of image processing).

## Conclusion

We developed an acoustofluidic scanning fluorescence nanoscope that provides superior resolution without sacrificing the field of view of the image. By combining the principles of acoustofluidics with microsphere-based microscopy, our platform overcomes the traditional trade-off between resolution and field of view observed with most conventional fluorescence microscopes^58,59^. The presence of a microsphere in the scanning fluorescence nanoscope led to stronger fluorescence compared to that without a microsphere, which provides significant utility in biological applications and nanoparticle examinations^60–63^. The bidirectional acoustofluidic scanning design allowed excellent freedom to control the scan of the microsphere. The dual-camera configuration enabled the collection of the fluorescent signal as well as the positional information of each microsphere to form an image with a large field of view. Finally, the image correction algorithm significantly reduced image distortion, resulting in a clearer and more accurate representation of the sample. Based on these features, the acoustofluidic scanning fluorescence nanoscope will be valuable for biomedical imaging and lab-on-a-chip systems and will facilitate the advancement of diagnostic tools^64^.

## Experimental section

### Optical characterization

As shown in Fig. 1c, we installed a CMOS camera (Zyla 4.2 Plus, Andor, USA) for fluorescence imaging and a CMOS camera (DFK 33UX264, Imagingsource, USA) for microsphere tracking on an upright microscope (BX51WI, Olympus, Japan) with a ×60 objective lens (NA: 0.7, Olympus, Japan). A white light source was combined with a blue bandpass filter (FL488-10, Thorlabs, USA) to act as an excitation source, and the fluorescence camera was combined with a green bandpass filter (FB530-10, Thorlabs, USA) to receive fluorescence from the sample. To capture both images simultaneously, we installed a 50:50 beam splitter (CCM1-BS013, Thorlabs, USA) at the intersection point between the two cameras.

### Fabrication of the acoustofluidic device

Two circular piezoelectric transducers (AB2720B-LW100-R, PUI Audio, Inc., USA) were bonded onto a 150 µm thick cover glass (24 × 50 mm C8181-1PAK, Sigma-Aldrich, USA) with epoxy bonding (PermaPoxyTM 5 min General Purpose, Permatex, USA). The distance between the two transducers was 6 mm.

### Microsphere preparation and experimental setup

To perform microsphere imaging, we chose 20 µm polystyrene microspheres (refractive index: 1.6, Sigma-Aldrich, USA). The microspheres were diluted with deionized water before being placed on the sample surface. To maintain a consistent water channel height between the device and sample, a square cover glass (#1.5, 10 × 10 mm, Ted Pella, USA) was placed at both ends of the device. MATLAB (version: R2021) script was designed and executed to control the function generator (FY6600, FeelTech, China) and CMOS cameras simultaneously. These cameras were used to collect the image data. Acoustic burst mode with 0.2 s intervals was applied, and image acquisition for the two cameras was executed every 0.2 s.

### Quantitative assessment of resolution enhancement

To quantitatively assess the resolution enhancement provided by the microsphere-assisted imaging technique, we compared images obtained with the microspheres (Fig. S6a, c, e) with those obtained without microspheres (Fig. S6b, d, f). This comparative analysis was focused on three representative cases within the same region of interest. Using the images collected, we measured the distance between pairs of 500 nm fluorescent nanoparticles in both the magnified (with microspheres) and nonmagnified (without microspheres) images. These measurements allowed us to calculate the magnification factors by dividing the magnified distance by the nonmagnified distance, yielding values of 2.235, 2.385, and 2.263, which substantiated the claim of superior resolution with this approach.

### Simulation of the acoustic field

To understand the acoustic energy distribution within the device, a model of an acoustic device was designed with COMSOL Multiphysics®. The model (Supplementary Fig. 1) included two piezoelectric transducers, a thin layer of epoxy, a cover glass, and water under the cover glass. The top boundary of the fluid domain was set to the impedance boundary of glass (density of 2230 kg/m^3^ and sound speed of 5602 m/s), and the other surrounding boundaries, i.e., the water layer, were set to the impedance boundary of air (density of 1.21 kg/m^3^ and sound speed of 343 m/s). All other boundaries were set as free boundaries to mimic real experiments. The simulation parameters and setup can also be found in Supplementary Fig. S5. The piezoelectric effect and acoustic structure boundary multiphysics interfaces were used to couple the electrostatic, solid mechanics, and pressure acoustics modules. A frequency domain study was used to visualize transducer excitation. A 2.1 kHz and 4*V*_PP_ signal was applied to one side of the transducer using the electrostatics module, and the other side was set to 0*V*_PP_. The acoustic pressure amplitude and arrows of the acoustic intensity were plotted to analyze the acoustic field, and the corresponding displacement distribution is also provided in Supplementary Fig. S4.

### Imaging sample preparation

To experimentally demonstrate the scanning performance of the system, we fabricated a fluorescent nanoparticle sample with a hard PDMS (PP2-RG07, Gelest, Inc., USA) membrane. The green fluorescent nanoparticle sample (200 nm: FSDG002, 500 nm: FSDG003, Bangs Laboratories, Inc., USA) was diluted with deionized water and loaded on the cover glass (24 × 50 mm C8181-1PAK, Sigma-Aldrich, USA). Then, the sample was dried at room temperature for 3–6 hours. After drying, we applied a hard PDMS mixture to the sample and ran a spin coating (WS-650-23, Laurell Technologies, USA) process. Then, the sample was baked at 60 °C for 30 min in an oven.

### Image processing

To generate the final image, the collected images were processed in the following order. First, a circle-finding algorithm was executed with the image from camera #2, as shown in the bottom panel of Fig. 1c, which stored information on the microsphere coordinates and radii. The magnification factor was calculated using the ratio of the sample grating line pitch length between camera #1 and camera #2. The calculated magnification factor (0.984) was then multiplied by the coordinates and radius and applied to the images from camera #1, as shown in the top panel of Fig. 1c. Next, the magnified circle images of the microspheres were cropped from the images of camera #1. Finally, the cropped images were pasted onto the final image with a lens distortion restoration technique to ensure matching between the images. Each image was processed recursively in the same manner. The final scanned image was generated with the repetitive image processing algorithm.

### Supplementary information

(Supporting information)Acoustofluidic fluorescence scanning nanoscopy with large field of view

Video SV1
