# Supplementary material for: Acoustofluidic scanning fluorescence nanoscopy with a large field of view
Source: Microsyst Nanoeng. 2024 May 10;10:59. doi: 10.1038/s41378-024-00683-8 (PMC11081950; doi:10.1038/s41378-024-00683-8)
Supplement: Supplementary file 1 — (Supporting information)Acoustofluidic fluorescence scanning nanoscopy with large field of view [file 41378_2024_683_MOESM1_ESM.docx]

Supporting Information

**Acoustofluidic scanning fluorescence nanoscopy with large field of view**

*Geonsoo Jin,^1†^ Neil Upreti,^2†^ Joseph Rich,^2^ Jianping Xia,^1^ Chenglong Zhao^3^* and Tony Jun Huang^1*^*

^1^ Thomas Lord Department of Mechanical Engineering and Materials Science, Duke University, Durham, North Carolina 27708, United States

^2^ Department of Biomedical Engineering, Duke University, Durham, NC 27708, United States

^3^ The MITRE Corporation, McLean, VA 22102, USA

^†^ These authors contributed equally to this work

*Email: czhao@mitre.org; tony.huang@duke.edu

**
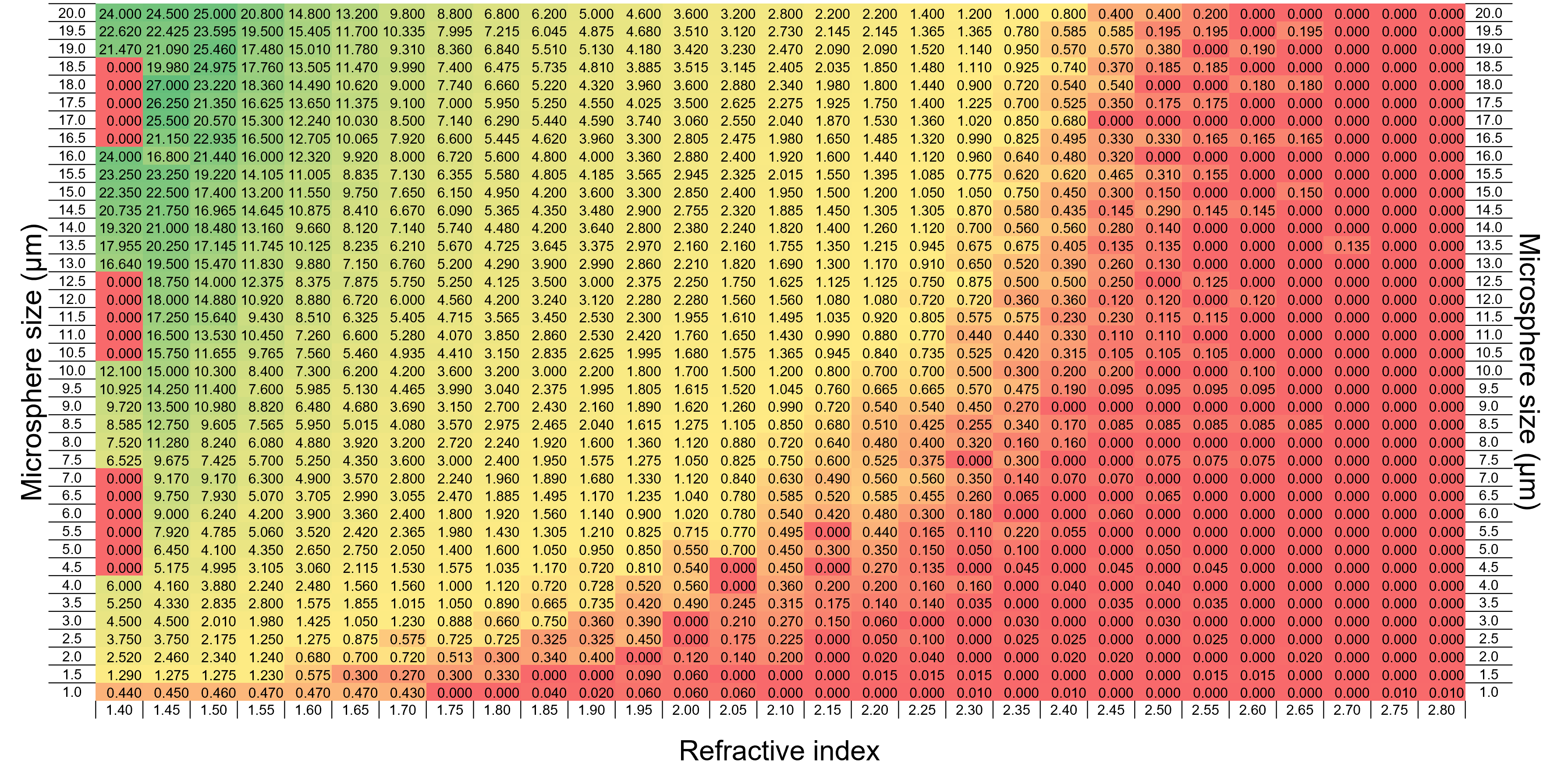
**

**Figure S1.** FEM simulation result of the focal distance map as a function of the microsphere diameter and refractive index.

**
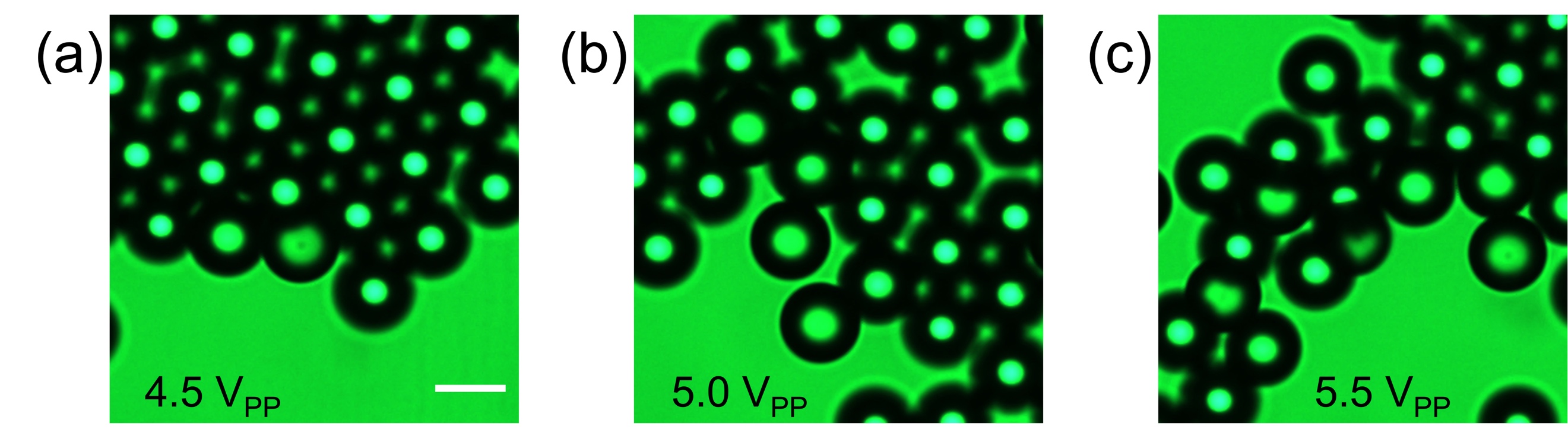
**

**Figure S2.** Microspheres floating when a voltage higher than 4 V_PP_ is applied from a function generator. (a) 4.5 V_PP_, (b) 5.0 V_PP_, and (c) 5.5 V_PP_. Scale bar is 20 µm.

**
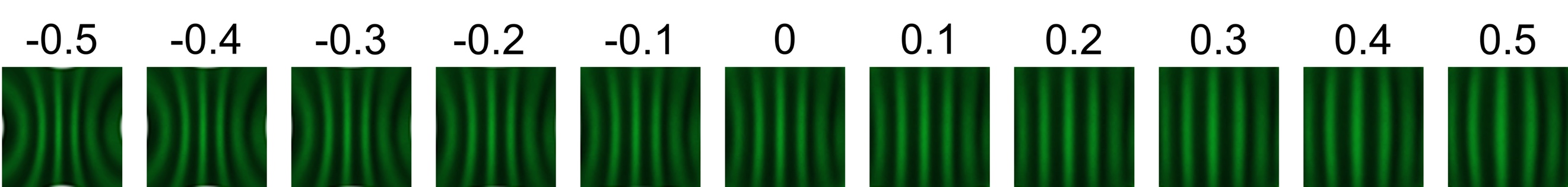
**

**Figure S3.** Line grating images by a microsphere with different distortion correction parameters.


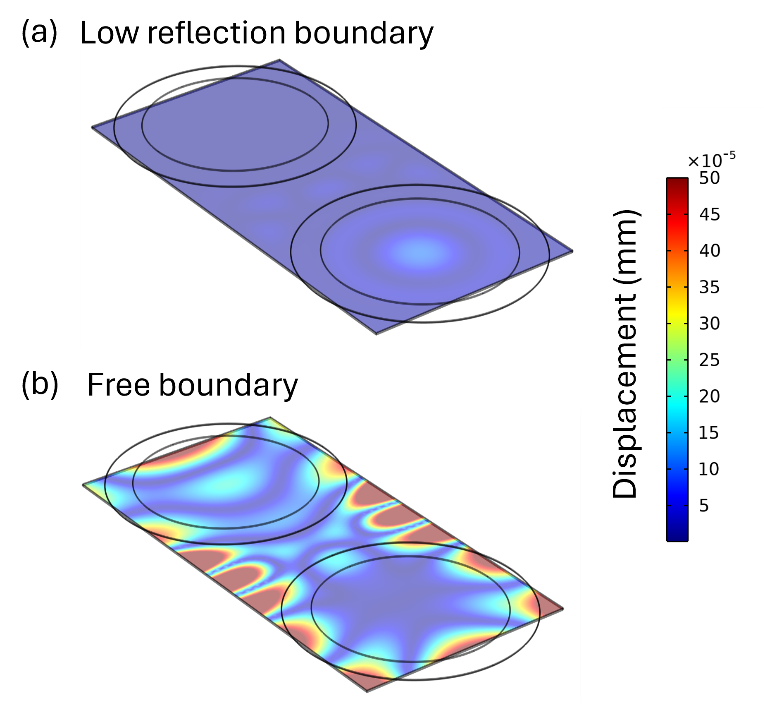


**Figure S4.** The displacement distribution in cover glass with (a) low reflection boundary and (b) free boundary. The strong and nonuniform distribution of displacement and acoustic pressure come from the boundary reflection of the device.


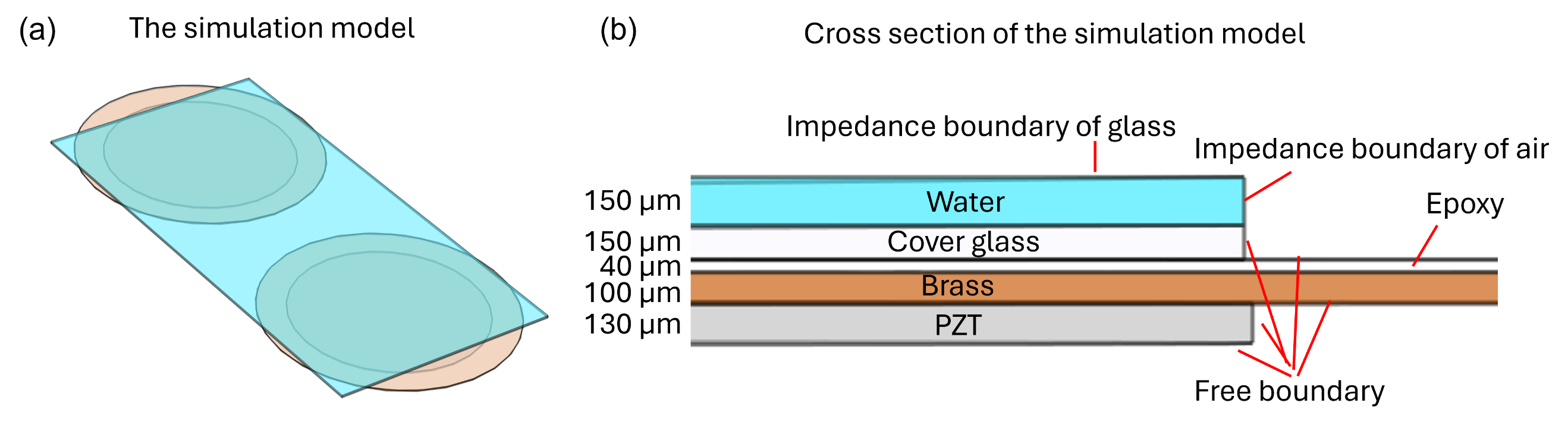


**Figure S5.** (a) The three-dimensional model used to calculate the displacement in cover glass and acoustic pressure in water. (b) The cross section of the simulation model shows the thickness of each layer and boundary conditions.


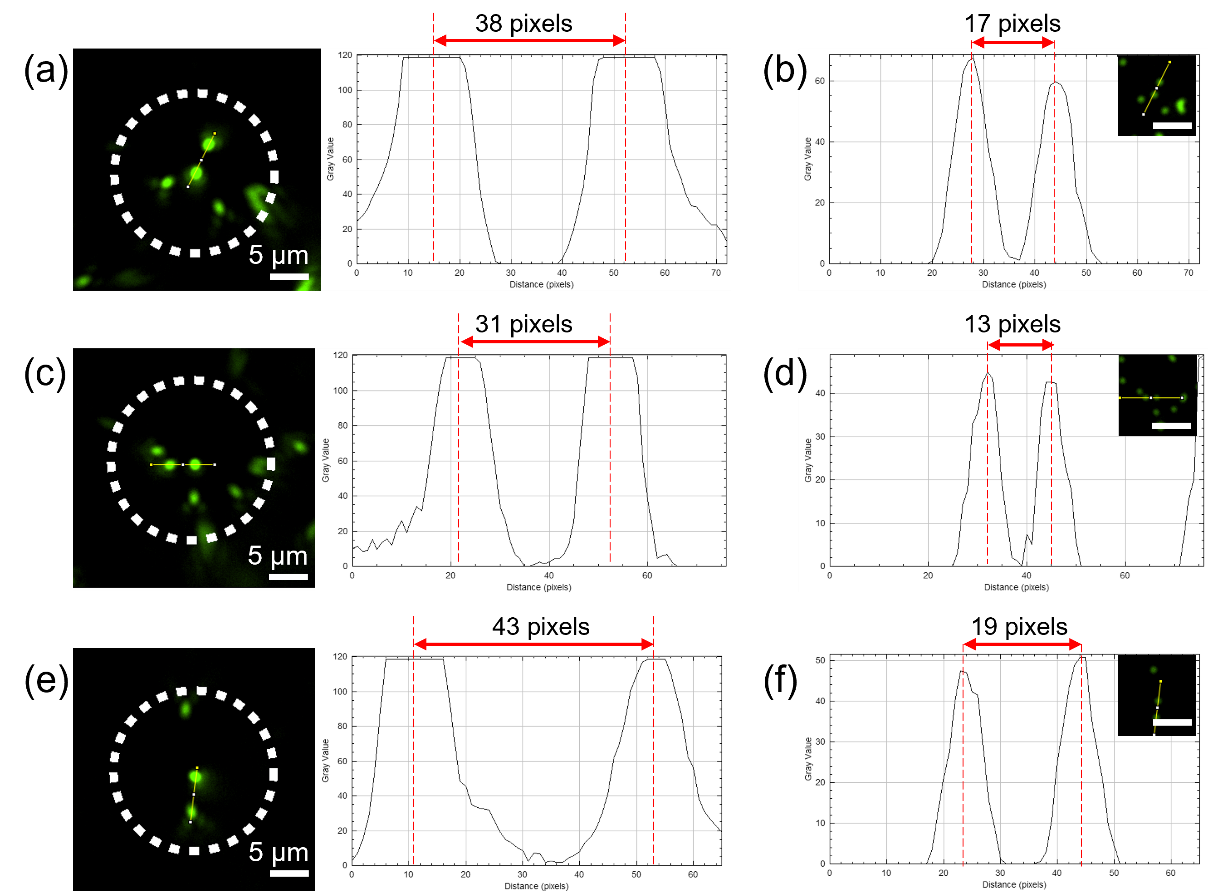


**Figure S6. Quantitative resolution comparison of the microsphere and non-microsphere conditions within the same region of interest.** (a, c, e) Microsphere conditions and (b, d, f) non-microsphere conditions with graphs showing the difference between magnified and unmagnified distance between two points. Scale bar: 5 µm.

**Video SV1.** Microparticle movement by the acoustofluidic scanning fluorescence nanoscope. We applied 4 V_PP_, 2.1 kHz, and 0.2 sec interval bursts to the acoustofluidic device.
